# The NGF Metabolic Pathway is altered in a Rat Model of Human‐like Tauopathy

**DOI:** 10.1002/alz70855_103151

**Published:** 2025-12-23

**Authors:** Sonia Do Carmo, Quentin Bonomo, Joshua T Emmerson, Ai Liu, Cynthia Sun, Chloé Levasseur, A. Claudio Cuello

**Affiliations:** ^1^ McGill University, Montreal, QC, Canada; ^2^ Oxford University, Oxford, United Kingdom

## Abstract

**Background:**

Cholinergic signaling is crucial for learning, memory, and attention. In Alzheimer's disease (AD) the degeneration of basal forebrain cholinergic neurons (BFCNs) is a key pathological feature driven by an impaired maturation of nerve growth factor (NGF). Our lab identified the NGF metabolic pathway, which regulates mature NGF (mNGF) availability, and demonstrated its dysregulation in human AD brain tissue and biofluids, as well as in amyloid pathology rat models. However, the impact of tau pathology—another AD hallmark—on cholinergic dysfunction is not well understood. This study aims to determine how tau pathology affects the NGF pathway and cholinergic function.

**Method:**

We examined NGF pathway proteins in cortical and hippocampal samples from homozygous McGill‐R955‐hTau transgenic rats, which model tauopathy. At 10 months, R955‐hTau rats show tau hyperphosphorylation and social behavior deficits. By 14 months, tau misfolding, aggregation, NFT‐like inclusions, glial activation, and cognitive impairments emerge. At 20 months, advanced tau pathology includes neuronal loss, brain atrophy, and myelin and vascular abnormalities. NGF pathway protein levels were assessed at 9, 14, and 20 months using Western blot and ELISA. Cholinergic bouton density in the parietal cortex of 20‐month‐old rats was assessed with a VAChT antibody, and BFCNs density and size were measured with a ChAT antibody.

**Result:**

In 20‐month‐old R955‐hTau rats with advanced tauopathy, we observed a significant increase in neuroserpin and proNGF levels, along with a decrease in mNGF levels. This NGF dysregulation was absent in younger rats, indicating that alterations in the NGF pathway occur later in tau pathology. Preliminary analyses also revealed disruptions in other NGF pathway components and reduced cortical VAChT immunoreactivity suggesting a consequential cholinergic atrophy.

**Conclusion:**

Our studies in McGill‐R955‐hTau rats would indicate that the advanced tauopathy provokes an additional dysregulation of the brain's NGF metabolic pathway leading to an exacerbated forebrain cholinergic atrophy.

